# Murine astrovirus infection course and antibody response in different mouse strains

**DOI:** 10.1038/s41684-025-01573-w

**Published:** 2025-06-26

**Authors:** Jessica Seib, Daniela Höfler, Lena Hornetz, Nicole Ohl, Katrin Götz, Klaus Vogel, Julia Butt, Katja Schmidt

**Affiliations:** 1 Microbiological Diagnostics, German Cancer Research Center, Heidelberg, Germany; 2 Infections and Cancer Epidemiology, German Cancer Research Center, Heidelberg, Germany; 3 Central Animal Laboratory, Center for Preclinical Research, German Cancer Research Center, Heidelberg, Germany

**Keywords:** Infectious-disease diagnostics, Virology, Viral infection

## Abstract

Monitoring the health of mice used in animal experiments constitutes an important instrument toward microbiological standardization, as infections can alter physiological parameters and immune reactions and may therefore have an essential impact on experimental outcome. In view of the high prevalence rates of murine astrovirus (MuAstV) infections in laboratory mouse facilities worldwide and the potential impact on research outcomes and reproducibility, there is a need to include MuAstV in the existing laboratory mice health monitoring programs. Here, to determine the sentinel strain and diagnostic method of choice, we aimed to assess the course of MuAstV infection and the resulting immune response in three immunocompetent mouse strains (Crl:CD1 (ICR), C57BL/6J and BALB/cOlaHsd) and one immunodeficient strain (NOD.Cg-*Prkdc*^*scid*^ *Il2rg*^*tm1Wjl*^/SzJ) by analyzing blood, feces and tissue samples with MuAstV-specific polymerase chain reaction and serology. Depending on the mouse strain, the duration of infection and viral load differed significantly, as well as the rapidity and quantity of antibody production. Virus shedding in immunocompetent mice was limited to a maximum of 4 weeks, whereas immunodeficient mice shed virus for the entire duration of this study. A fast antibody response with high titers was found only in outbred CD1 mice. In C57BL/6J and BALB/c mice, however, seropositivity and high antibody levels were reached only after the second infection. These results not only improve our understanding of the infection characteristics of MuAstV, presumably the most prevalent virus in laboratory mice, but also help to set up a health monitoring routine giving meaningful and reliable results on the MuAstV infection status of laboratory mouse populations.

## Abstract (web summary)

This study analyzes the course of murine astrovirus infection and the immune response in multiple mouse strains to identify the best sentinel strain and the best diagnostic method when including murine astrovirus in health monitoring programs.

## Main

Astroviruses are small, nonenveloped, single-stranded RNA viruses that belong to the family *Astroviridae* and were first described in 1975 following an outbreak of diarrhea in children^1^. Seroprevalence studies indicate that astrovirus infections in humans are extremely common with a rate of infection of more than 90% in children at the age of 5 years and 70% in immunocompetent healthy adults^2–6^. As a result of advances in next-generation sequencing and pathogen discovery, astroviruses have also been isolated from a large and still growing variety of animal species^7–9^.

Astrovirus infections in mice (*Mus musculus*) were first described in 1985 by Kjeldsberg and Hem^10^. Murine astroviruses (MuAstVs) were of little or no interest until 2011 when MuAstV was rediscovered in feces of a wild mouse by metagenomic analysis and shortly thereafter in laboratory mouse colonies of research facilities and commercial breeders^11–13^. In 2018, a novel, highly divergent MuAstV was found in wild house mice near New York City by analysis of the fecal virome^14^. It was designated MuAstV-2, while all other previously found astroviruses in laboratory mouse colonies of research facilities and commercial breeders were classified as MuAstV-1 (referred to as MuAstV throughout this Article). Shortly after, MuAstV-2 was also detected in sentinel mice of a laboratory animal facility and found to be a contaminant of a murine T helper cell line used for antigen production^15^.

Although so far there have been no reports of pathogenicity even in highly immunodeficient mouse strains^13,16–18^, there is increasing evidence that MuAstV infection affects the cell-specific innate immune response and alters the interferon baseline level. These effects can then influence experimental results, for example, in microbiome and infection studies or in studies targeting the immune response^19,20^. In this context, the inclusion of MuAstV testing in routine health monitoring in laboratory animal facilities should be considered. This raises the question of which sentinel strain is appropriate and which diagnostic method is best suited regarding the time point of testing. Due to the asymptomatic course of infection, MuAstV infection is usually confirmed by reverse transcription polymerase chain reaction (RT-PCR) of fecal material. A bead-based multiplex MuAstV immunoassay for serological detection was also recently published^16^.

The aim of this study was to assess the course of MuAstV infection with respect to (1) local and systemic infection, potential spreading in the host, (2) duration of virus shedding and virus elimination, (3) the threshold for detection of measurable specific antibody titers, (4) immune protection against reinfection by another MuAstV isolate and (5) differences of the infection course in widely used wild-type mouse strains. Three strains of immunocompetent mice and one immunodeficient mouse strain were infected orally with MuAstV. Organ samples, blood and feces were collected periodically and analyzed by RT-PCR, quantitative (q)RT-PCR and multiplex MuAstV serological assay. Results will help to improve the understanding of MuAstV biology and infection characteristics in different mouse strains that, in turn, will facilitate identifying the sentinel strains and diagnostic methods of choice.

## Results

### Primary sites of MuAstV replication and virus shedding

**Fig. 1:**
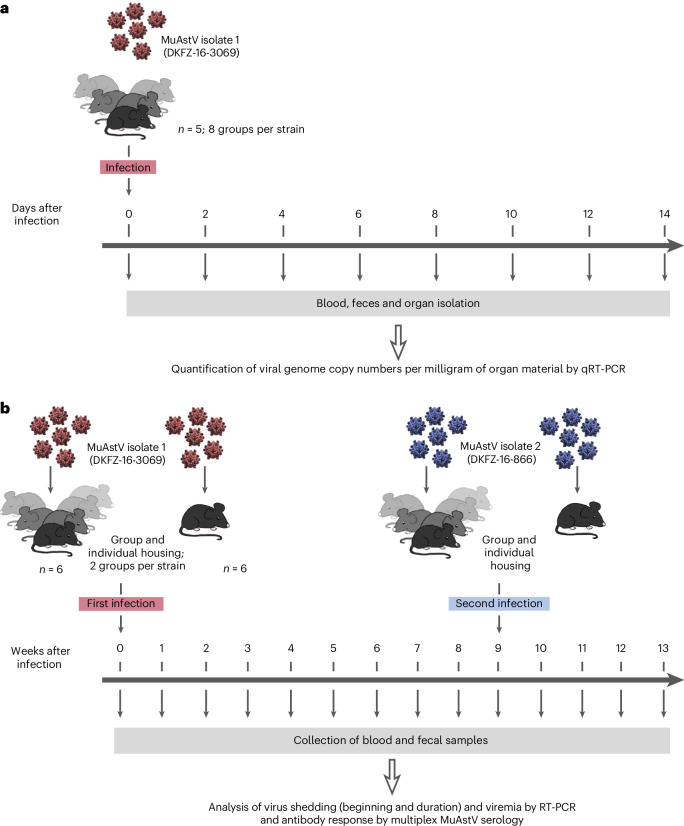
Schematic illustration of the study design for the short-term and long-term experiment. **a**, Short-term experiment: seven groups of five mice per mouse strain were infected with MuAstV isolate 1 (one control group per strain was not infected and immediately analyzed). For every examination day (2, 4, 6, 8, 10, 12 and 14 dpi), one group per strain was euthanized for blood, feces and organ isolation. **b**, Long-term experiment: for each strain, two groups of six mice (one group-housed and another group of six individually housed mice) were infected with MuAstV isolate 1. Every week, blood and fecal samples were collected for subsequent analysis.

In two experimental parts, a short-term and a long-term experiment, mice of three immunocompetent strains and one immunodeficient strain, that is, CD1, C57BL/6, BALB/c and NOD scid gamma (NSG), were infected to analyze the course of the MuAstV infection. The aim of the short-term experiment was to quantitatively analyze the viral load in various organs, feces and blood within 2 weeks of infection, whereas the aim of the long-term experiment (13 weeks duration) was to examine the duration of virus shedding and viremia, as well as to analyze the antibody response (Fig. 1).

To characterize the replication kinetics of MuAstV infection over a 2-week period (short-term experiment), mice of the different strains were orally infected with MuAstV isolate 1 and virus levels in the intestinal tract, feces, extragastrointestinal organs and blood were analyzed. Samples from five mice per strain were collected on day 0 (negative control) and every second day post infection (dpi) over the 2-week period and assayed by quantitative RT-PCR (qRT-PCR) (Fig. 1a). Viral load was calculated as the genome copy numbers per milligram of organ sample (Supplementary Dataset 1).

**Fig. 2:**
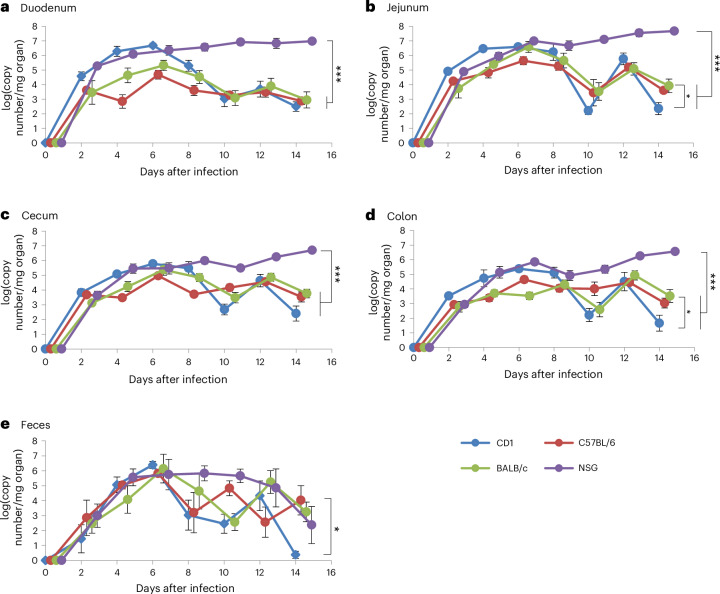
MuAstV replication kinetics in four different segments of the intestine and feces over 14 dpi. **a**–**e**, The viral load, detected by qRT-PCR, is plotted as genome copy number (*y* axis, log_10_-transformed) over 14 days (*x* axis) in the duodenum (**a**), jejunum (**b**), cecum (**c**), colon (**d**) and feces (**e**). The four mouse strains are pictured in blue (CD1), red (C57BL/6J), green (BALB/c) and purple (NSG). For visual clarity, the data points in the figure are shown with a small spatial offset to avoid data points or curves hiding each other. Mean values of five animals per strain and time point as well as standard errors are depicted. *P < 0.05, ***P < 0.001; Tukey test (*n* = 5 per time point).

Statistical analysis by two-way analysis of variance (ANOVA) revealed a highly significant effect of the mouse strain and time point after infection on the viral load in all intestinal sections examined (*P* < 0.001 in each case; Supplementary Table 1). MuAstV RNA was already detectable in the intestine at 2 dpi (Fig. 2). In the immunocompetent mice, virus replication reached a maximum at 6 dpi regardless of mouse strain and decreased steadily thereafter. Viral loads in jejunum and duodenum were the highest, with 5 × 10^6^ RNA copies per milligram of organ material in CD1 mice, and 4 × 10^5^ and 4 × 10^6^ copy numbers in C57BL/6J and BALB/c mice, respectively (Fig. 2a,b). The viral load in the large intestine (cecum and colon) was slightly lower (Fig. 2c,d). By contrast, the viral load of immunodeficient NSG mice increased steadily for the entire duration of the experiment and reached maximum values of 5 × 10^7^ in jejunum and 5 × 10^6^ in cecum at 14 dpi. Despite high viral loads, no pathological findings or symptoms were observed in any of the immunocompetent or the immunodeficient mice.

Virus shedding with feces also started at 2 dpi. In CD1, BALB/c and C57BL/6J mice, the amount of excreted virus reached a maximum at 6 dpi and decreased afterward (Fig. 2e). The amount of excreted virus in NSG reached its maximum around 4 dpi and remained constant until 10 dpi before the excretion declined. At the peak of virus shedding (6 dpi), no significant differences in the viral load between the mouse strains were detected (one-way ANOVA *F*_3,16_ = 0.176; *P* = 0.911). However, regarding all time points, the mouse strain had a significant effect on the amount of virus in the feces (*P* = 0.023; Supplementary Table 1). Remarkably, the amount of virus excreted in feces showed a considerable variation between individual animals (for example, 14 dpi, CD1: standard deviation (s.d.) = 0.52 log(genome copy numbers per mg organ material); C57BL/6J: s.d. 2.19; BALB/c: s.d. 1.48; NSG: s.d. 2.77) (Fig. 2e).

### Systemic MuAstV infection

**Fig. 3:**
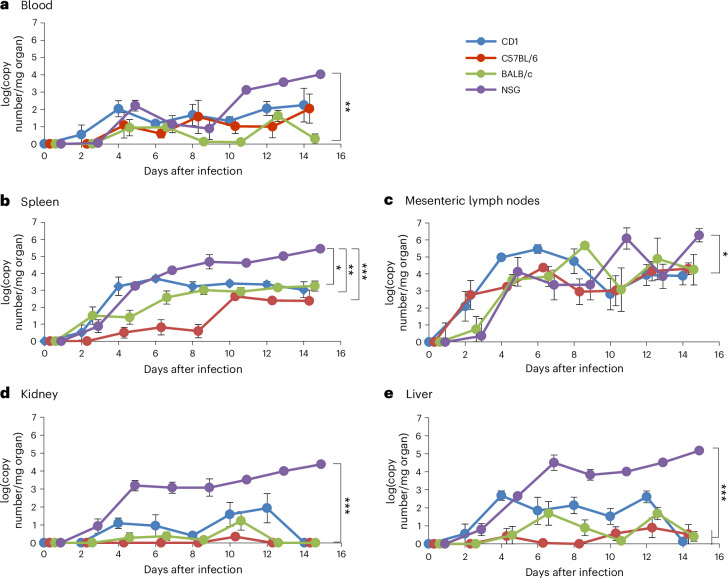
Viral load in blood and extraintestinal organs over 2 weeks after infection with MuAstV. **a**–**e**, The course of viral load, detected by qRT-PCR, is plotted as genome copy number (*y* axis, log_10_) over a period of 14 days (*x* axis) in the blood (**a**), spleen (**b**), mesenteric lymph nodes (**c**), kidney (**d**) and liver (**e**). The four mouse strains are pictured in blue (CD1), red (C57BL/6J), green (BALB/c) and purple (NSG). For visual clarity, the data points in the figure are shown with a small spacial offset to avoid data points or curves hiding each other. Mean values of five animals per strain and time point as well as standard errors are depicted. *P < 0.05, **P ≤ 0.01, ***P < 0.001; Tukey test (*n* = 5 per time point).

To clarify if MuAstV infection is localized to the intestine or if MuAstV induces a systemic infection, virus RNA levels in the blood and in the extragastrointestinal organs were analyzed. As shown in Fig. 3a, the first detection of virus RNA in the blood happened 2 days later than in the intestine or feces. In immunocompetent mouse strains, the viral load in the blood was low with a maximum of 2 × 10^2^ genome copy numbers at 12 and 14 dpi. In 10% of CD1, 43% of C57BL/6J and 47% of BALB/c mice between 4 dpi and 14 dpi, the virus did not enter the blood circulation or was below the detection limit (Supplementary Dataset 1). On the contrary, all NSG mice were systemically infected and showed a continuous increase in the viral load in blood over the 2-week experiment with a maximum of 10^4^ copy numbers at 14 dpi.

Furthermore, analysis of viral loads in the immune organs showed that at 2 dpi MuAstV RNA was detectable in the mesenteric lymph nodes and spleen of all mouse strains (Fig. 3b,c). In NSG mice, a continuous increase in viral load was observed in spleen and lymph nodes over the period of the study. In the immunocompetent mouse strains, viral loads increased until day 6 or 8 after infection and then reached a plateau. While the viral load in liver and kidney in the immunocompetent mouse strains was overall low and decreased by 14 dpi, the viral load in the immunodeficient mouse strain was highest at the end of the 2-week period (Fig. 3d,e).

In addition to MuAstV quantification in the organs mentioned above, the viral load in the urinary bladder, the heart, lung and brain between 0 dpi and 8 dpi was analyzed comparing CD1 and NSG mice (Supplementary Fig. 1). Viral RNA was detected in all of the organs, although in the heart and lung the viral loads in NSG were comparably higher than in CD1 mice (two-way ANOVA, main factor strain; heart *F*_1,20_ = 14.12, *P* < 0.001; lung *F*_1,20_ = 60.06, *P* < 0.001). Most remarkably, MuAstV was also found in the brain, as has been described for humans and other animal species, with maximum copy numbers of almost 10^4^ in CD1 and 2.5 × 10^4^ in NSG mice.

### Duration of MuAstV shedding after first and second infection

**Fig. 4:**
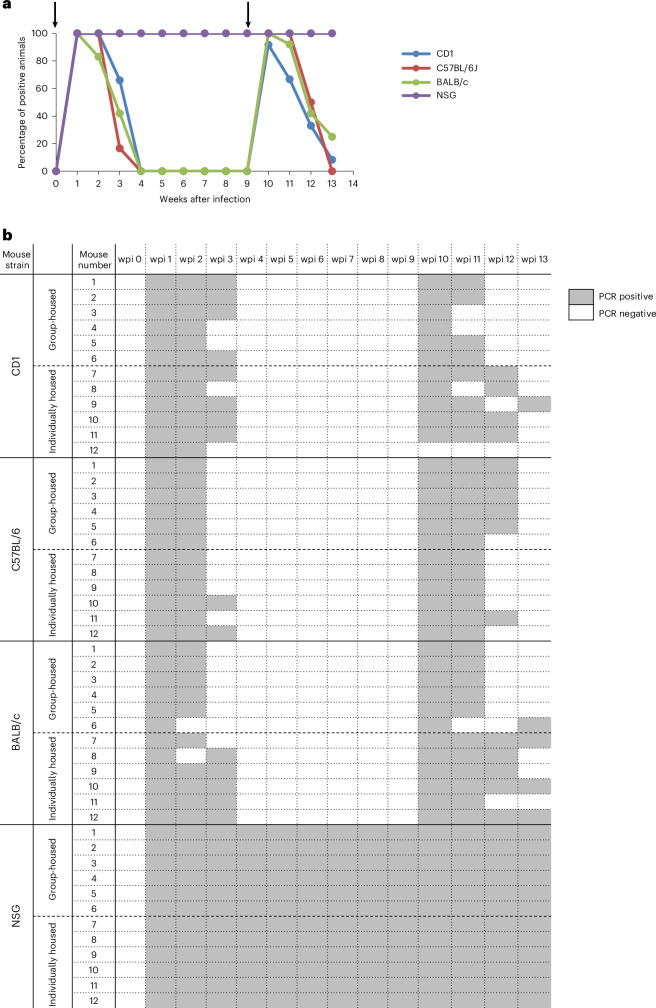
Duration of MuAstV shedding in feces, detected by RT-PCR, after first and second infection over 13 weeks (long-term experiment). **a**, The percentage of virus-positive mice among 12 mice per mouse strain (*y* axis) is plotted against wpi over 13 weeks (*x* axis). CD1 in blue, C57BL/6J in red, BALB/c in green and NSG in purple. Arrows indicate the first infection at 0 wpi and second infection at 9 wpi (*n* = 12). **b**, The onset and duration of virus shedding is shown for each individual animal.

The overall objective of the 13-week long-term experiment was to characterize the course of MuAstV infection in individual mice of four different strains (Fig. 1b). Duration of virus shedding in feces and viremia were assessed by RT-PCR (Supplementary Dataset 2). For each strain, six animals were either group-housed or kept individually in separate cages to clarify whether mice that have already eliminated the virus can be reinfected by having contact with other mice still shedding the virus. As no or only minor differences in the duration of shedding after infection were found between the animals kept in a group and the ones kept alone (Mann–Whitney test, CD1 mice: *U*_1_ = 18, *P* = 1.0; C57BL/6J: *U*_1_ = 12, *P* = 0.39; BALB/c: *U*_1_ = 5, *P* = 0.04; NSG: *U*_1_ = 18, *P* = 1.0), the results of all mice for each strain were used for reasons of presentation and comparison (see below and Fig. 4).

Analysis of the duration of shedding in all immunocompetent mouse strains (*n* = 6 individually housed mice) revealed that there was no significant effect of the strain on the duration of shedding (Kruskal–Wallis test, *H*_2_ = 1.7, *P* = 0.427). As expected, all mice of the four strains started MuAstV shedding in feces within 1 week after infection (wpi) (Fig. 4). Virus shedding in immunocompetent mouse strains lasted for 3–4 weeks. At 4 wpi, all immunocompetent mice had eliminated the virus. This was in clear contrast to the immunodeficient NSG mice, which excreted MuAstV for the entire duration of the experiment.

At 9 wpi, 5 weeks after the first MuAstV infection was cleared, all immunocompetent mice were reinfected with the heterologous MuAstV isolate 2, simulating conditions where different virus isolates circulate within the same mouse population. All immunocompetent animals except one CD1 mouse were reinfected and repeatedly shed the virus (Fig. 4b). Overall, the duration of shedding in feces after the first and second infection was similar with a maximum duration of 4 weeks, although at 13 wpi one CD1 mouse and three BALB/c mice were still RT-PCR positive (Fig. 4). NSG mice shed the virus for the entire duration of the experiment and were consequently not reinfected.

**Fig. 5:**
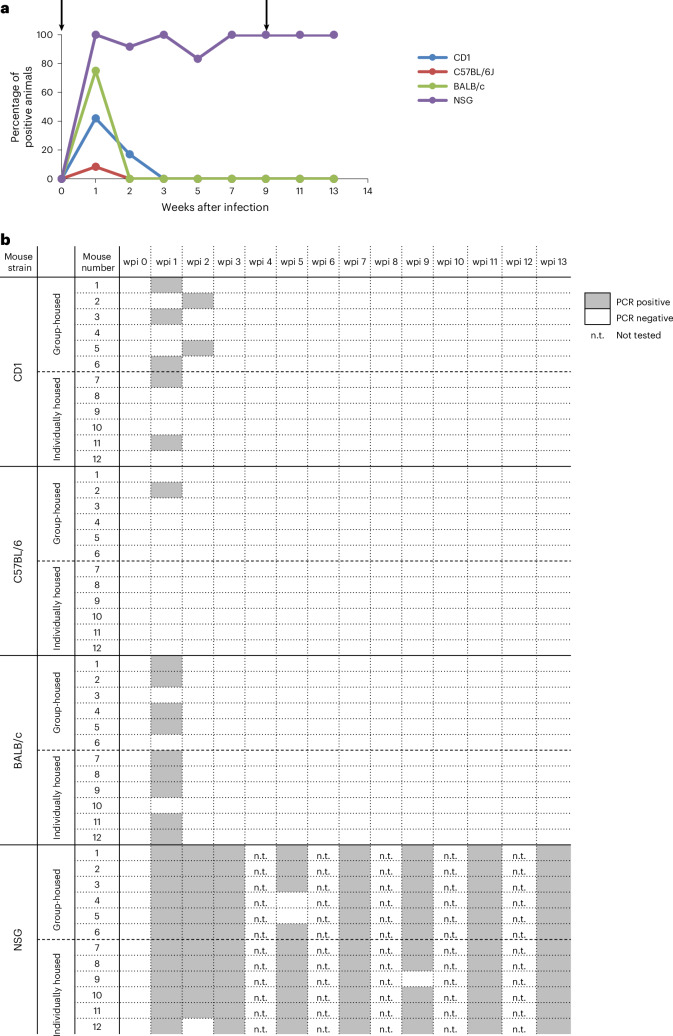
Number of viremic mice, detected by RT-PCR, at different time points after MuAstV infection over 13 weeks. **a**, The percentage of virus-positive mice among 12 mice per mouse strain (*y* axis) is plotted against wpi over a period of 13 weeks (*x* axis). CD1 in blue, C57BL/6J in red, BALB/c in green and NSG in purple with 12 mice per strain. Arrows indicate the first infection at 0 wpi and a second infection at 9 wpi (*n* = 12). **b**, The onset and duration of virus shedding is shown for each individual animal.

Consistent with the findings of the short-term experiment, viremia was found not only in immunodeficient but also in immunocompetent mice. Most remarkably, all NSG mice became viremic within a week after infection and viremia persisted for the entire duration of the experiment (Fig. 5). By contrast, viremia in the immunocompetent strains was short and only some animals became viremic. At 1 wpi, virus was found in the blood of 58% of CD1, 75% of BALB/c and 8% of C57BL/6J mice (Supplementary Dataset 3). After the second infection with a heterologous MuAstV isolate at 9 wpi, none of the immunocompetent mice became viremic.

### MuAstV-specific immune response

**Fig. 6:**
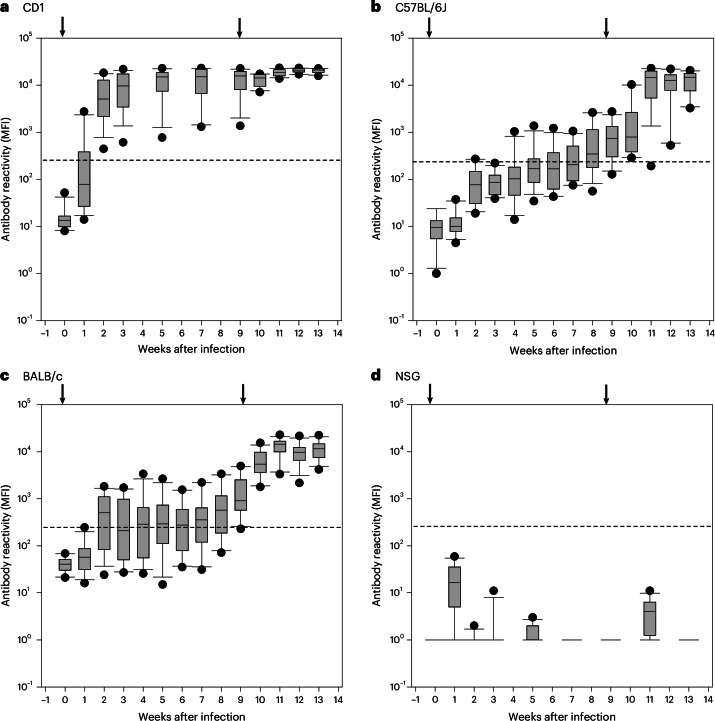
Immune response of different mouse strains after first and second MuAstV infection. **a**–**d**, MFI values of 12 mice per strain (CD1 (**a**), C57BL/6J (**b**), BALB/c (**c**) and NSG (**d**)) were analyzed by multiplex MuAstV serology (*y* axis) and plotted against weeks after infection (*x* axis) in a semilogarithmic format as boxplots. Boxplots are confined by the first and third quartile (interquartile range, Q1–Q3) in which 50% of data are found. Whiskers show the 10th and 90th percentile. The line within each boxplot represents the median. The threshold of 300 MFI is displayed as a dashed line. Data below the threshold are considered negative, whereas data above the threshold are considered positive. Arrows indicate the first and second infection (0 and 9 wpi).

Before MuAstV infection and every week for 13 consecutive weeks, antibody levels were measured by multiplex MuAstV serological assay (Supplementary Dataset 4). Analysis of the median fluorescence intensity (MFI) values, averaged for each mouse strain (*n* = 6 individually housed mice) and time point (*k* = 11 weeks, that is, without three missing weeks in strain CD1, because due to high MuAstV antibody titers blood samples were taken only every second week to minimize burden of animals without additional scientific information) by two-way ANOVA revealed that the mouse strain and the time point (wpi) have a highly significant effect on the specific antibody levels (two-way ANOVA, main effect mouse strain *F*_2,165_ = 40.34, *P* < 0.001; main effect time point *F*_10,165_ = 48.83, *P* < 0.001; Supplementary Table 1). Significant differences in antibody levels (average MFI values of each mouse over all time points) were found in CD1 mice compared with the two other immunocompetent strains C57BL/6J and BALB/c (Tukey test: CD1 versus C57BL/6J and BALB/c; *F*_2,165_ = 40.34; *P* < 0.001). For reasons of presentation, Fig. 6 shows the combined data of individually housed and group-housed mice as there were no statistically significant nor relevant differences in means nor in the s.d. of MFI values between these groups (for example 9 wpi, Brown–Forsythe test, CD1 mice: s.d. group-housed 5,507, s.d. individually housed 6,921, *F*_1,10_ = 0.818, *P* = 0.389; C57BL/6J: s.d. group-housed 520, s.d. individually housed 956, *F*_1,10_ = 0.442, *P* = 0.521; BALB/c: s.d. group-housed 1,634, individually housed 1,652, *F*_1,10_ = 0.073, *P* = 0.792). All CD1 mice were clearly seropositive with a median MFI value of 5,000 already 2 wpi. After 5 wpi, a median MFI value of 15,000 and, after second infection, maximum MFI values of 21,000 were reached. By contrast, antibody levels of C57BL/6J and BALB/c increased slowly, and 9 weeks after the first MuAstV infection, median antibody levels of only 700 and 900 MFI, respectively, were measured. Around 25% of C57BL/6J and 10% of BALB/c mice did not even exceed the cutoff for seropositivity (Supplementary Dataset 4). High MuAstV-specific antibody levels, which were clearly above the cutoff for all animals tested, could be detected only after reinfection with MuAstV isolate 2. As expected and due to their lack of B cells, immunodeficient NSG mice did not produce any antibodies.

## Discussion

This study compares the course of MuAstV infection in four different mouse strains by determining viral loads in different organs, blood and noninvasive fecal samples and by quantifying antibody levels. The results of our study are intended to contribute to a better understanding of the course of infection and the spread of a virus that is often overlooked and, so far, not widely included in health monitoring programs.

In line with a previous study, which suggested that the virus is highly infectious^16^, all mice of this study were successfully infected, followed by virus shedding in feces within 2 days after infection. Successful experimental infection of mice at different ages, including pups and adult mice between 3 and 12 weeks of age, has been reported in the literature before^13,17,21,22^. In accordance with these studies, the highest viral loads were found in the gastrointestinal tract of mice, especially in the small intestine, which serves as the primary site of MuAstV replication^19,23^. Virus RNA was also found in the immune organs, spleen and mesenteric lymph nodes, and in kidney and liver, although to a much lower extent. Because the genome copy numbers in liver and kidney were relatively low, it cannot be stated with certainty whether parenchymal infection and replication occurred or whether virus RNA was detected in blood residues in these organs, as the mice were not perfused before organ harvesting. Altogether, there were obvious differences between mouse strains during MuAstV infection that were dependent on the immune status: while immunocompetent animals reached maximum viral loads in the intestine 6 dpi and viral loads declined afterward, in immunodeficient NSG mice, the viral load increased over the entire duration of the experiment. In another study. the MuAstV isolate reached the highest virus levels in the intestinal tract between 8 and 13 dpi and levels did not drop before 17 dpi^22^, suggesting there could be MuAstV isolate-specific differences in the infection and replication kinetics. In our study, in all immunodeficient mice and in most, but not all, immunocompetent mice, MuAstV infection was shown to cause systemic infection. These results may have direct consequences for tissue and organ isolation and retransplantation experiments. Although it is generally assumed that the fecal–oral route of transmission is similar across species, there are many reports that astrovirus infections in other species are not limited to the gastrointestinal tract and that the virus is able to spread beyond, causing extragastrointestinal astrovirus-associated disease in animals and humans^24–31^.

Although the amount of virus shedding within feces was found to be similar in immunocompetent and immunodeficient mice, the duration of shedding was substantially different. The immunocompetent mouse strains of this study excreted MuAstV over a maximum period of 4 weeks, in contrast to the immunodeficient NSG that excreted the virus for the entire duration of this study (13 weeks), suggesting a long-term or even persistent infection of mice with severe defects in the immune system, in line with earlier reports^16,21,22^. Due to long-term or even persistent virus shedding, immunodeficient mice may pose a great risk for virus transmission to naive, so far uninfected animals.

Duration of virus shedding for up to 4 weeks in our study differed somewhat from other infection studies, in which MuAstV was found in the feces for up to 5.5 weeks in CD1 mice^17^, and 3 and 10 wpi of C57BL/6 mice with two different MuAstV isolates^22^. As astroviruses are a diverse group of dissimilar viruses, due to a high mutation rate often found in RNA viruses, it is plausible that the features, or the strain of an astrovirus isolate determine the infection characteristics and the duration of infection and shedding. Likewise, MuAstVs show a considerable diversity, as seen by numerous published partial genome sequences isolated from wild and laboratory mice^11,12,21,22,32^, which in consequence may result in isolate- or strain-specific differences in virus tropism and persistence.

Since the rediscovery of the virus, primarily molecular testing of feces, if at all, has been applied to check the MuAstV infection status of mouse colonies. As MuAstV has so far not been added to the list of infectious agents recommended for testing by the Federation for European Laboratory Animal Science Associations (FELASA)^33^, testing for past and ongoing MuAstV infections is not routinely done and little effort has been taken to expand the palette of diagnostic methods. Although serological assays that allow antibody testing have been offered recently by some commercial diagnostic laboratories, very little is known about the virus-specific antibody response. In our study, we analyze the humoral immune response of experimentally MuAstV-infected mice in the course of an infection using a multiplex MuAstV serological assay. In conclusion, the antibody levels differed greatly depending on the mouse strain tested. A fast response with high antibody levels was found only in outbred CD1 mice, whereas in C57BL/6J and BALB/c mice, antibody levels increased only slowly over weeks and were often barely above the threshold. Only after a second infection with another MuAstV isolate were antibody levels substantially increased and high antibody levels reached. We also found that MuAstV-specific antibodies did not protect against reinfection with a heterologous MuAstV isolate several weeks after clearance of the first infection. In this study, we did not examine if the antibodies were protective against reinfection with the same MuAstV strain. Cortez et al.^22^ also showed a successful reinfection with a homologous isolate 8 and 12 weeks after the clearance of the initial infection. Results of our study point in the same direction, as group-housed and single-housed mice showed no differences in the duration of MuAstV shedding (Fig. 4). Obviously group-housed mice that had already cleared the first infection earlier did not get reinfected by having contact with cage mates still shedding the virus, indicating that there is a short refractory period after MuAstV clearance. The general inability of MuAstV-specific antibodies to protect against reinfection was also shown earlier in our seroprevalence study^16^. High seroprevalence rates of up to 100% did not result in low numbers of virus-positive animals, as the virus prevalence rates were still comparably high. Due to a lack of sustained immunity, astroviruses can circulate freely within the population, and mice can be infected several times in their life, as no age-related virus prevalence was observed. Likewise, experiments with turkey astrovirus demonstrated that antibodies are not essential in controlling an astrovirus infection^34^. Most remarkably in our study, no MuAstV was found in the blood of the immunocompetent mice after the second infection in contrast to the first infection. It is therefore conceivable that, even though MuAstV-specific antibodies do not prevent reinfection and virus shedding, antibodies can prevent viremia and the spread of MuAstV to extraintestinal organs via the blood circulation and, consequently, a systemic course of infection.

Because no statistically significant nor relevant differences were found between group-housed and individually housed mice of the long-term experiment, we assumed that this also applied to the short-term infection experiment and that the five mice within a cage behaved nearly independently to give a sample size of *n* = 5. Due to animal welfare reasons, we felt obliged to house animals for the short-term experiment together in groups. However, we are aware of this potential shortcoming regarding the statistical evaluation.

In the course of this study, no gross pathological lesions nor symptoms, which may be associated with experimental infection, were observed in any of the immunocompetent or the immunodeficient mice. The lack of pathogenicity and the absence of clinical symptoms, such as diarrhea or fecal inconsistency, during experimental MuAstV infections were also reported by others^17,18,22^. Although MuAstV does not cause symptomatic disease in mice, it is conceivable that subclinical infections alter physiological parameters and immune phenotypes. Thus, differences in the MuAstV infection status of mice may contribute to altered experimental outcomes or to increased interindividual variations, so that larger numbers of animals are needed to yield statistically significant results. In fact, recently, Ingle and colleagues demonstrated that chronic MuAstV infection complements defects in the adaptive immunity by elevating IFN-λ in the intestinal epithelial barrier in immunodeficient mice, which provides protection against other enteric viruses^20^. They concluded that persistent infection in immunodeficient mice results in immunologically distinct baseline IFN responses, which may affect experimental outcomes. In general, there is increasing evidence that the microbiota affects disease pathogenesis in humans and animals and that the microbiota of animal models may have an essential impact on experimental outcome^35–38^. Several studies indicate that astroviruses have the ability to alter the microbiome diversity and composition. For example in poultry, astrovirus infections cause enteritis mortality syndrome, which is associated with an outgrowth of atypical *E. coli*^39,40^. In humans, astrovirus infections in healthy children were found to cause the greatest decrease in bacterial diversity in comparison with other gastrointestinal viral pathogens, affecting in particular members of the genus *Bifidobacterium*, which are considered an important component of the healthy microbiome^41,42^. Similarly, Cortez et al. observed a decrease in bacterial diversity at the peak of infection and a disruption of the bacterial composition in the MuAstV model^43^. In view of high prevalence rates of MuAstV in mouse colonies, the high diversity of MuAstVs and the high chance of reinfections, the probability of interference with other murine models of disease has to be considered.

The results of this study underline the importance of carefully selecting not only the mouse strain used as sentinel but also the time point of testing and the testing method for monitoring the astrovirus infection status. We showed that commonly used immunocompetent mouse strains, such as C57BL/6 and BALB/c, excrete the virus for a few weeks only, making molecular testing of feces not reliable. A negative PCR result states the absence of an acute MuAstV infection but gives no information about past MuAstV infections or within the population monitored. Although the infection is often cleared within a few weeks, the antibodies often remain for a long time. Hence, serological methods are widely used to detect viral infections for health monitoring of laboratory animals. Likewise, serological methods are better suited for a reliable detection of a MuAstV infection in a population. The multiplex MuAstV serological assay of this study allows the sensitive and specific detection of current or past MuAstV infection and is well suited to be used in the health monitoring of rodent colonies owing to its multiplex format and high-throughput testing capacity. However, the inclusion of serological testing methods in the evaluation of infection characteristics showed that not every strain is equally suited in their use as sentinel mice. It became clear that the antibody levels of certain mouse strains are not sufficiently high after a single round of infection, so that serological testing is not conclusive and that a repeated infection is necessary to produce high antibody levels that make the unequivocal detection of infections in the populations possible. Therefore, the type of housing must be considered for the evaluation of serological testing results. In units with mice housed in open cages, facilitating MuAstV circulation, high seroprevalence rates and high antibody levels due to repeated MuAstV infections can be found, compared with units with mice housed in individually ventilated cages, which hampers virus transmission. While outbred mouse strains are better suited to monitor the MuAstV status of a population by serology, immunodeficient mice should be preferred for testing with molecular methods owing to long-term or permanent shedding of the virus.

In conclusion, the results of this study add to the current knowledge of MuAstV infection in different mouse strains, which is important not only for the selection of sentinel strains and diagnostic methods but also for the selection of the genetic background of mice for their use as models in astrovirus pathogenesis studies in human medicine research. Given that the microbiological quality of laboratory animals is gaining increasing importance, that (undetected) infections may directly affect experimental variability and research outcomes in animal experimentations, and that MuAstV has been shown to induce changes in the microbiome composition and immune reactions, the inclusion of MuAstV testing into existing health monitoring programs is indicated.

## Methods

### Animals

The mouse strains used in this study (internal designations: CD1, C57BL/6J, BALB/c and NSG) were bred as gnotobiotic (Taconic Altered Schaedler flora (ASF) associated) mice for biotechnical and health monitoring purposes and housed under high hygiene conditions in the central breeding unit at the vivarium of the German Cancer Research Center (DKFZ) in Heidelberg. CD1 (official strain nomenclature Crl:CD1 (ICR); Crl strain code 022), C57BL/6J (TJL stock no. 000664) as well as NSG (NOD.Cg-*Prkdc*^*scid*^ *Il2rg*^*tm1Wjl*^/SzJ; TJL stock no. 005557) and BALB/c (BALB/cOlaHsd) mice were purchased from Charles River, The Jackson Laboratory and Harlan, respectively. To ensure genetic stability according to genetic standards, the inbred variants were rederived after approximately 10 generations of DKFZ breeding corresponding to about 20 generations of total breeding after separation of DKFZ and original strain. The CD1 outbred stock was usually only expanded at DKFZ by crossing CD1 individuals born in the vivarium of DKFZ with CD1 refresher partners obtained by mating of original Crl animals and freezing and rederiving the resulting embryos. In rare cases where no such refresher animals were available, a further expansion step was performed at DKFZ to ensure sufficient CD1 animals.

Mice of both sexes between 6 and 9 weeks of age were housed in individually ventilated cages (GM500, Greenline, Tecniplast). All cage beddings (aspen material), nesting material (aspen wood, 24–120 mm, Abedd Vertriebs GmbH), food (Mouse Maintenance No. 3437, KLIBA NAFAG) and water were autoclaved before use. Cage changing was done under the laminar flow hood. For infection and sampling, animals were handled in a biosafety 2 cabinet. New overgloves were used after all mice of the same strain were handled and 0.5% Wofasteril classic (Kesla Hygiene AG) was used for disinfection after working processes.

To maintain the high hygienic status of animals in the central breeding unit, intensive routine hygiene monitoring was conducted by testing contact sentinels and colony animals weekly for viral and bacterial infections and parasites. Microbiological testing included all infectious agents listed by the recommendations of the FELASA plus encephalomyocarditis virus (species *Cardiovirus rueckerti*), MuAstVs, yeasts and all aerobic-growing bacteria^33^. Microbiological testing of animals was conducted in the Microbiological Laboratory of the Center for Preclinical Research at the DKFZ using bacteriological methods, multiplex serology, immunofluorescence, enzyme-linked immunosorbent assay, hemagglutination inhibition assay, conventional PCR, qPCR, multiplex PCR and microscopy. Until performing infection experiments of this study, the hygiene status of the central breeding unit had not changed.

The animal facility of the DKFZ has been officially approved by the responsible authority (Regional Council of Karlsruhe, Germany) under the official approval file no. Az 35-9185.64BH DKFZ. Housing conditions are thus in accordance with the German Animal Welfare Act (TierSchG) and the EU Directive 2010/63/EU. Compliance with institutional guidelines and legal regulation regarding care and handling of animals was ensured by designated veterinarians according to Article 25 of Directive 2010/63/EU and Animal-Welfare Body according to Article 27 of Directive 2010/63/EU. Animal testing in the frame of this study was officially approved by the local governmental authorities (Regional Council of Karlsruhe, Germany) under the notification number G-196/16 (approval date 21 November 2016).

### Infection and sample collection

Two MuAstV RT-PCR positively tested mice from two different astrovirus-positive units at the DKFZ were selected as donor mice for the preparation of MuAstV containing fecal suspension. MuAstV isolate 1 (DKFZ-16-3069; GenBank accession number MW072508) originates from a Rag2-knockout mouse and MuAstV isolate 2 (DKFZ-16-866; GenBank accession number MW072509) was obtained from a NSG mouse (Supplementary Fig. 2). Samples were also tested by RT-PCR to confirm the absence of mouse norovirus, the only other virus present in these units, and by qPCR to confirm the absence of mouse kidney parvovirus. Feces were suspended in sterile-filtered PBS and centrifuged for 10 min at 3,700*g* and 4 °C. The supernatant was then sterile-filtered using a 5-µm and a 0.8-µm and 0.2-µm syringe filter, followed by 16S rRNA PCR to check the absence of bacterial contaminations (primers A and H, published in ref. ^44^). The 1% fecal suspensions of the two isolates were prepared for the oral infection, and aliquots were kept at −80 °C until use.

#### Short-term experiment (2 weeks)

The short-term experiment was run over 14 days including 8 examination days. Each examination day, a group of five mice per mouse strain was analyzed. At 0 dpi, one group per strain was not infected and immediately dissected (control mice). The remaining seven groups per strain were orally infected with 100 µl of a 1% MuAstV isolate 1 containing fecal suspension (2.6 × 10^8^ genome copies), and every second dpi one group per mouse strain was euthanized for sample collection (Fig. 1a). Blood was collected during dissection by cardiac puncture after euthanasia with CO_2_. After opening the abdominal wall, the spleen, left kidney, part of the left hepatic lobe, mesenteric lymph nodes and urinary bladder as well as parts of duodenum, jejunum, cecum and colon were isolated under sterile conditions. Fecal samples were collected from the rectum or anus. In addition, the heart, left lung and brain were removed for further analysis. During dissection, inner organs and tissues were checked for gross pathological abnormalities. The absence of infections with other aerobic- or facultative anaerobic-growing bacteria than those of the ASF was rechecked by bacterial culture. All samples were kept at −80 °C before analyzing by qRT-PCR.

#### Long-term experiment (13 weeks)

Before infection (0 wpi) blood and feces were collected and analyzed as baseline samples. All mice of the long-term experiment were orally infected with 100 µl of a 1% MuAstV isolate 1 (2.6 × 10^8^ genome copies) containing fecal suspension. After the infection, mice were kept for a duration of 13 weeks. Fecal and blood (from the tail vein) samples were collected weekly and analyzed by RT-PCR and multiplex serology (Fig. 1b). In case of a high MuAstV antibody titer (MFI >1,000), blood samples were collected only every second week to minimize the burden to the animals without additional scientific information. Per mouse strain, two groups of mice with six animals each were infected: one group of mice was housed together (group-housed) and animals of the other group were kept individually. According to the experimental design, mice were reinfected once tested MuAstV negative by RT-PCR for at least five consecutive weeks. Mice were reinfected with 100 µl of a 1% fecal suspension containing a heterologous MuAstV isolate 2 (genome copy numbers not determined because the MuAstV qPCR was designed for specific MuAstV isolate 1 detection). Fecal samples and blood were analyzed by RT-PCR and gel electrophoresis. Antibody response was analyzed by multiplex MuAstV serology.

### RNA preparation

RNA was extracted using the Maxwell 16Lev device and the Maxwell 16Lev simplyRNA Tissue Kit (Promega GmbH). Ten milligrams of sample was added to 200 µl of homogenization solution. The sample was mechanically homogenized by a sample homogenizer (Precellys24, Bertin Instruments) at 5,000*g* for 15 s. A total of 200 µl lysis buffer was added to the homogenate before the mixture was transferred into the first well of the Maxwell processing cartridge. For the following steps, the manufacturer’s instructions were followed and the RNA was eluted in 50 µl RNAse-free water.

### RT-PCR

The RT-PCR included a two-step PCR: 1 μl of isolated RNA was transcribed into cDNA using the QuantTect Reverse Transcriptase (QIAgen) and subsequently applied to PCR using the Multiplex PCR kit (QIAgen). Both kits were used according to the manufacturer’s instructions with half of the reaction volume. The primers used for PCR to detect both MuAstV isolates were previously published by Ng et al.^32^. One microliter of cDNA was added to the reaction mixture. Reaction conditions and the appropriate annealing temperatures for primers (61 °C) were adjusted according to the QIAgen manual supplied. Amplicons were analyzed by ethidium bromide gel electrophoresis.

### qRT-PCR and RNA standard

For the quantification of MuAstV copy numbers in the short-term experiment, one-step qRT-PCR was performed by using the iTaq Universal Probes One-step Kit (Bio-Rad) according to the manufacturer’s instructions. For primer design, multiple alignment of published MuAstV sequences was used, including strain STL 1 (GenBank accession no. JX544743.1), strain STL2 (accession no. JX544744.1) and strain BSRI1 (accession no. KC609001.1), together with sequenced MuAstV isolates present in the DKFZ. MuAstV-specific primers named qFW_MuAstV_ORF1b (5′-CACATGTACCRGTGGTAT-3′; position 3637-3654 (s)) and qBW_MuAstV_ORF1b (5′-TCRAATGCCTGAAGCCA-3′; position 3788-3772 (as)) and the probe qP2_MuAstV_ORF1b (5′-FAM(6-carboxyfluorescein)-CACCGYTACACCGTGYTGC-BBQ(BlackBerry Quencher)-3′; position 3670-3688 (s)) target a 152-bp fragment within the RNA-dependent RNA-polymerase gene. Five microliters of RNA was added to the reaction mixture. Reaction conditions and the appropriate annealing temperature for the primers (60 °C) were adjusted according to the Bio-Rad manual. The qRT-PCR was run with a LightCycler 480 instrument (Roche Diagnostics), and the LightCycler 480 software LCS480 1.5.0.39 was used for analysis with the settings ‘Second derivative maximum method’ and ‘High confidence’.

To generate a synthetic RNA to be used as standard, the target sequence within the RNA-dependent RNA polymerase gene was synthesized on the basis of MuAstV isolate 1 (DKFZ-16-3069) (Eurofins Genomics Germany) and cloned into the vector pBluescript II KS (Stratagene). To obtain linear RNA, the vector was linearized with EcoRI-HF, reverse transcribed using the T7 RNA-polymerase (Thermo Fisher Scientific) and treated with the RNase-free DNase set (QIAgen) to remove plasmid DNA following the manufacturer’s instructions. RNA was purified using the RNeasy Mini Kit (QIAgen), and the concentration was measured with a NanoDrop 1000 spectrophotometer (Thermo Fisher Scientific). The copy number was calculated and serial dilutions ranging from 10^8^ to 10^0^ molecules (genome copies) per microliter were used to construct the calibration curve by plotting mean quantification cycle (Cq) values against the calculated RNA copy number. Copy numbers were calculated by absolute quantification using an RNA standard. One-step qPCR efficiency was 90% (*R*^2^ = 0.9994), and the detection limit was between 10 and 100 RNA copies per reaction. The viral loads of the virus solution used for experimental infection (MuAstV isolate 1) and mouse samples analyzed were calculated as genome copy numbers per milligram of organ sample.

### Multiplex MuAstV serology

Multiplex MuAstV serology was performed as described previously^16^. In brief, sera were diluted 1:5 in HA buffer (90 mM NaCl, 3.45 mM NaH_2_PO_4_ and 7 mM Na_2_HPO_4_, pH 7.2), before being further diluted 1:50 in preincubation buffer. The assay was run as a two-plex using recombinantly expressed MuAstV VP27 and an IgG control. A Luminex analyzer (BioPlex200, Bio-Rad Laboratories) was used to simultaneously detect the bead set and to quantify the amount of bound serum antibody by a secondary antibody (biotin-SP-conjugated AffiniPure goat anti-mouse IgG + IgM (H + L) by Jackson ImmunoResearch, 1:1,000 diluted in blocking buffer) and reporter fluorescence (streptavidin-R-phycoerythrin, MOSS, 1:500 diluted in blocking buffer). The number of antibodies bound to the antigen was reported as the MFI of at least 75 beads per antigen per measure. Samples were defined as positive if the net MFI values were above the cutoff of 300 MFI^16^.

### Statistical analysis

Measured values were log-transformed to normalize data within groups and to stabilize variances. ‘No ct’ values were set to log (viral copy numbers per mg organ material) zero. Transformed data did not differ significantly from normal distribution in 311 out of 353 cases over all variables (combinations of 4 strains × 8 sampling days × 10 organs (short-term experiment), and of 3 strains × 11 sampling weeks (long-term experiment); David test, *P* > 0.05)^45^. Due to the balanced design (*n* = 5 or *n* = 6 in all groups) the two-way ANOVA is robust against deviations from normality and variance homogeneity. Hence, a parametric two-way ANOVA was used to analyze main effects (mouse strain; time point after infection; *n* = 5 in all groups of the short-term experiment or *n* = 6 in all groups of the long-term experiment) and interactions (Supplementary Table 1). The Tukey test was applied as a post-hoc test with a focus on differences among mouse strains at time point 14 dpi. SigmaPlot version 14.0 (Systat Software) was used for statistical analyses.

### Reporting summary

Further information on research design is available in the Nature Portfolio Reporting Summary linked to this article.

## Online content

Any methods, additional references, Nature Portfolio reporting summaries, source data, extended data, supplementary information, acknowledgements, peer review information; details of author contributions and competing interests; and statements of data and code availability are available at 10.1038/s41684-025-01573-w.

## Supplementary information

Supplementary Information Supplementary Figs. 1 and 2 and Table 1.

Reporting Summary

Supplementary Data 1 Spreadsheet with viral loads given in log(viral copy number per milligram of organ material) for comparison of MuAstV replication kinetics in different organs and mouse strains over 14 days after infection (short-term experiment). These data were used to generate Figs. 2 and 3 and Supplementary Fig. 1.

Supplementary Data 2 Spreadsheet with RT-PCR results from fecal samples of mice to compare duration of MuAstV shedding in different mouse strains over 13 weeks after MuAstV infection (long-term experiment). These data were used to generate Fig. 4.

Supplementary Data 3 Spreadsheet with RT-PCR results from blood samples of mice to compare duration of MuAstV viremia in different mouse strains over 13 weeks after MuAstV infection (long-term experiment). These data were used to generate Fig. 5.

Supplementary Data 4 Spreadsheet with net MFI values for comparison of the antibody response in different mouse strains over 13 weeks after MuAstV infection (long-term experiment). These data were used to generate Fig. 6.

## Data Availability

All data supporting the findings of this study are available within the Article and its Supplementary Information. The sequences of the MuAstV isolates have been deposited in the NCBI Genbank with accession numbers MW072508 and MW072509.
