# Supplementary material for: Murine astrovirus infection course and antibody response in different mouse strains
Source: Lab Anim (NY). 2025 Jun 26;54(7):178–87. doi: 10.1038/s41684-025-01573-w (PMC12213600; doi:10.1038/s41684-025-01573-w)
Supplement: Supplementary file 1 — Supplementary Figs. 1 and 2 and Table 1. [file 41684_2025_1573_MOESM1_ESM.pdf]

lab animal

Supplementary information

https://doi.org/10.1038/s41684-025-01573-w

Murine astrovirus infection course and antibody response in different mouse strains

In the format provided by the authors and unedited

Supplementary information - Murine astrovirus infection course and antibody response in different mouse strains

Jessica Seib, Daniela Höfler, Lena Hornetz, Nicole Ohl, Katrin Götz, Klaus Vogel, Julia Butt and Katja Schmidt

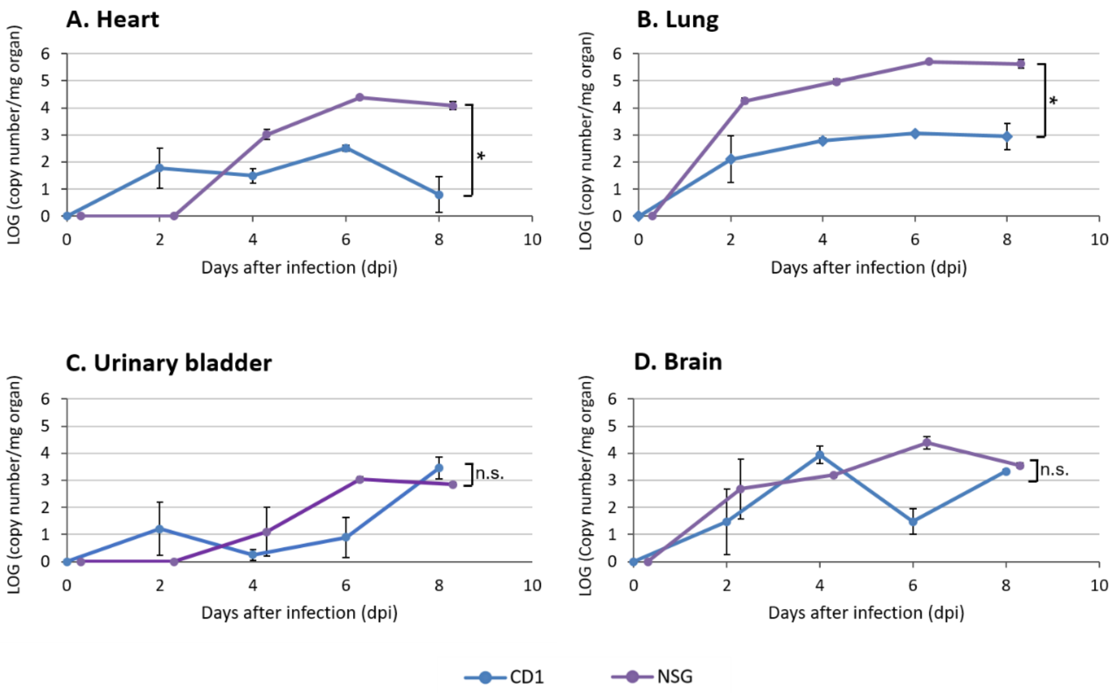

Supplementary Fig 1: Viral load in extra-intestinal organs heart, lung, urinary bladder and brain over 14 days after infection with MuAstV. Course of viral load in A. heart, B. lung, C. urinary bladder, and D. brain is plotted as genome copy number (y-axis, $\log_{10}$) over a period of 8 days (x-axis). Mouse strain CD1 is pictured in blue and NSG in purple. For reasons of visual clarity, the data points in the figure are shown with a small spacial offset to avoid that data points or curves hide each other. Mean values of three animals per strain and time point as well as standard errors are depicted. Significance is denoted by asterisks [(*) $p < 0.05$; n.s. not significant; Tukey-Test (n=3/time point)].

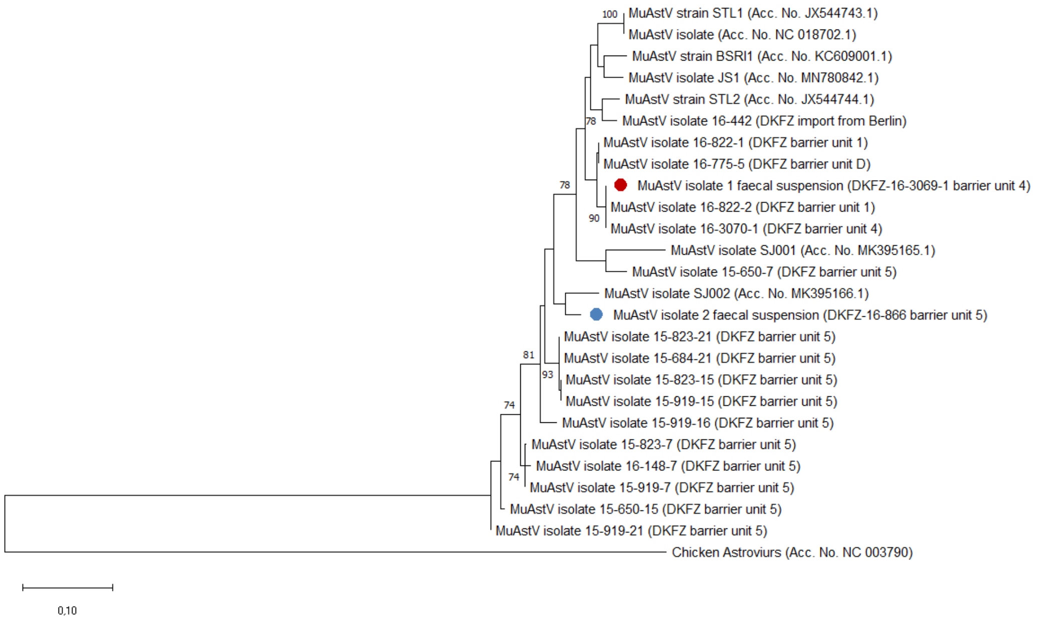

Supplementary Fig 2: Phylogenetic tree of diverse MuAstV isolates based on parts of the viral RNA-dependent RNA-polymerase gene (396 bp). The dots indicate DKFZ MuAstV isolate 1 (red) and isolate 2 (blue) used in this study which are set in relation to other already published MuAstV representatives (BSRI1, STL1, STL2, SJ001 and SJ002) and isolates from mice housed in different units at the DKFZ. A chicken astrovirus isolate was used as an out-group. The phylogenetic and molecular evolutionary analysis were conducted using MEGA X software [46]. The tree was constructed using a maximum-likelihood method and is supported by the Tamura 3-parameter model [47]. Figures at nodes indicate maximum likelihood bootstrap values of 500 runs under the specified model. Only higher values than 70 are shown. The scale bar represents 0.1 nucleotide (nt) substitutions per nt position.

Supplementary Table 1: Effect of mouse strain and time point after infection (dpi; wpi) on viral load (short-term experiment) and antibody level (long-term experiment) in different organs/samples – statistical analysis

		Two-way ANOVA					Effect size
	**Organ**	**Source of Variation**	**DF**	**MS**	**F**	***p***	**Eta squared (η^2)**
Viral load	Duodenum	Mouse Strain	3	50.91	66.62	<0.001	0.20
		Days post infection (dpi)	7	61.00	79.82	<0.001	0.56
		Mouse Strain x dpi	21	4.35	5.69	<0.001	0.12
		Residual	128	0.76			
		Model					0.87
	Jejunum	Mouse Strain	3	28.05	44.94	<0.001	0.10
		Days post infection (dpi)	7	84.53	135.43	<0.001	0.69
		Mouse Strain x dpi	21	4.92	7.88	<0.001	0.12
		Residual	128	0.62			
		Model					0.91
	Caecum	Mouse Strain	3	15.28	42.57	<0.001	0.08
		Days post infection (dpi)	7	59.01	164.37	<0.001	0.73
		Mouse Strain x dpi	21	3.06	8.53	<0.001	0.11
		Residual	128	0.36			
		Model					0.92
	Colon	Mouse Strain	3	18.22	35.15	<0.001	0.10
		Days post infection (dpi)	7	52.36	101.02	<0.001	0.64
		Mouse Strain x dpi	21	3.92	7.56	<0.001	0.14
		Residual	128	0.52			
		Model					0.88
	Faeces	Mouse Strain	3	10.19	3.30	0.02	0.03
		Days post infection (dpi)	7	68.48	22.19	<0.001	0.47
		Mouse Strain x dpi	21	5.07	1.64	0.05	0.11
		Residual	128	3.09			
		Model					0.61
	Blood	Mouse Strain	3	14.01	14.54	<0.001	0.14
		Days post infection (dpi)	7	12.65	13.13	<0.001	0.29
		Mouse Strain x dpi	21	2.43	2.52	<0.001	0.17
		Residual	128	0.96			
		Model					0.60
	Spleen	Mouse Strain	3	37.27	79.90	<0.001	0.24
		Days post infection (dpi)	7	35.55	76.21	<0.001	0.52
		Mouse Strain x dpi	21	2.62	5.62	<0.001	0.12
		Residual	128	0.47			
		Model					0.87
	Lymph node	Mouse Strain	3	1.29	0.63	0.60	0.01
		Days post infection (dpi)	7	55.33	27.08	<0.001	0.50
		Mouse Strain x dpi	21	5.50	2.69	<0.001	0.15
		Residual	128	2.04			
		Model					0.66

	Two-way ANOVA						Effect size
	**Organ**	**Source of Variation**	**DF**	**MS**	**F**	***p***	**Eta squared (η^2)**
	**Kidney**	Mouse Strain	3	62.10	149.93	<0.001	0.54
		Days post infection (dpi)	7	6.56	15.83	<0.001	0.13
		Mouse Strain x dpi	21	2.92	7.05	<0.001	0.18
		Residual	128	0.41			
		Model					0.85
	**Liver**	Mouse Strain	3	66.78	119.70	<0.001	0.44
		Days post infection (dpi)	7	13.37	23.97	<0.001	0.21
		Mouse Strain x dpi	21	4.36	7.81	<0.001	0.20
		Residual	128	0.56			
		Model					0.844
**Antibody level**	**Blood**	Mouse Strain	2	622136701	40.34	<0.001	0.11
		Weeks post infection (wpi)	10	752988946	48.83	<0.001	0.64
		Mouse Strain x wpi	20	23903283	1.55	0.071	0.04
		Residual	165	15421896			
		Model					0.78
